# Should Complex Cognitive Functions Be Mapped With Direct Electrostimulation in Wide-Awake Surgery? A Network Perspective

**DOI:** 10.3389/fneur.2021.635439

**Published:** 2021-04-12

**Authors:** Guillaume Herbet

**Affiliations:** ^1^Institute of Functional Genomics, INSERM, CNRS, University of Montpellier, Montpellier, France; ^2^Gui de Chauliac Hospital, Montpellier University Medical Center, Montpellier, France

**Keywords:** intraoperative cognitive mapping, electrostimulation, glioma, awake surgery, network architecture

## Introductive Remarks

It is now well-established that wide-awake neurosurgery with electrostimulation mapping is a safe technique for removing cerebral tumors drastically (1, 2), whilst sparing central, lowly compensable pieces of the anatomo-functional architecture (3, 4). In the last 10-year period, significant efforts have been made to implement new behavioral paradigms in the operating theater with the aim of giving patients the best opportunities to quickly recover after surgery and to resume a normal socio-professional life. Without being fully exhaustive, this includes tasks probing semantic (5, 6) and social cognition (7–9), motor (10, 11), and spatial cognition (12, 13), reading (14), and working memory (15). In turn, neuroscientic knowledge gained from multifunctional electrostimulation mapping procedures has been accumulating over the years in at least three directions: (i) the neuroplasticity potential of neurocognitive networks, (ii) the interindividual variability in the neural implementation of functional systems and (iii), the role of the main white matter tracts in different forms of cognition [for a review, see Herbet and Duffau (16)]. Overall, this growing knowledge is vital not only to help neurosurgeons better anticipate the surgery's functional outcomes (and thus better improve patient care), but also to continuously refine the way patients are operated on based upon valid neuroscientific foundations. In this respect, the virtuous circle that constitute the reciprocal interactions between neurosurgery and cognitive neuroscience (17) should be considered as the cornerstone on which to orient our decision-making regarding the ongoing debate on what kind of functions should be monitored *on-line* during awake procedures (18). This reflection is fully justified as wide-awake neurosurgery is now proposed much earlier for oncological purpose, and patients' survival is considerately longer than a couple of years ago. In this context, we have to set higher expectations with respect to preserving functions and to maintaining quality of life. But where to place the cursor for cognitive mapping without losing touch with the onco-functional balance (i.e. the best trade-off between extent of resection vs. preservation of functions) (19)? In this opinion article, I give some balanced perspectives on this debate, especially on the issue whether complex or flexible cognitions and behaviors (e.g., adaptive, multidetermined cognitions such as contextual decision-making or fast learning) can be reliably mapped given the network architecture on which they rest.

## Can Highly Distributed Vs. Modular Neural Systems Be Actually Mapped With Direct Electrostimulation?

It is certainly not new to say that the main functional systems of the human brain are physically rooted in a web of interconnected neurons, which are structured in the form of well-organized neural networks (20). These networks, however, differ significantly in terms of modularity, as a function of the wide range of more or less complex cognitions and behaviors they are supposed to underlay (21). It is indeed known that basic sensorimotor processes are supported by highly modular and local networks, whereas higher-order but still modality-specific or domain-specific cognitive functions are rather supported by distributed networks of cortical areas within which neural information needs to be integrated locally in each cortical node forming the network as well as globally between each node of the network. On a third level, goal-directed and flexible cognitions (resulting in complex behaviors) rest on the instantiation of transient and context-sensitive functional meta-systems that reflect specific patterns of between-network coordination, the functional integration of which is permitted by cortical hubs with a high degree of centrality (such as for e.g., the posterior part of the dorsolateral prefrontal cortex or the posterior dorsal cingulate cortex) (16, 22). Such highly integrative functioning is probably essential in the human ability to form new and creative behaviors, to efficiently perform cognitive-demanding activities, and to learn complex abilities. For example, learning a simple motor task necessitates the engagement of the sensorimotor, the attention, the visual and the executive networks, the sensori-motor system becoming sufficient to perform the task when automaticity is reached after several sessions of training (23). Different behavioral parameters seems to constrain the recruitment of this kind of highly distributed processing, in particular the complexity of the behavior task to be performed (complex materials necessitates intervention of domain-general networks, such as of the attention, executive, and working memory networks), the goal-directed vs. effortless nature of the task and the recruitment of conscious vs. unconscious processing (24).

**Figure 1:**
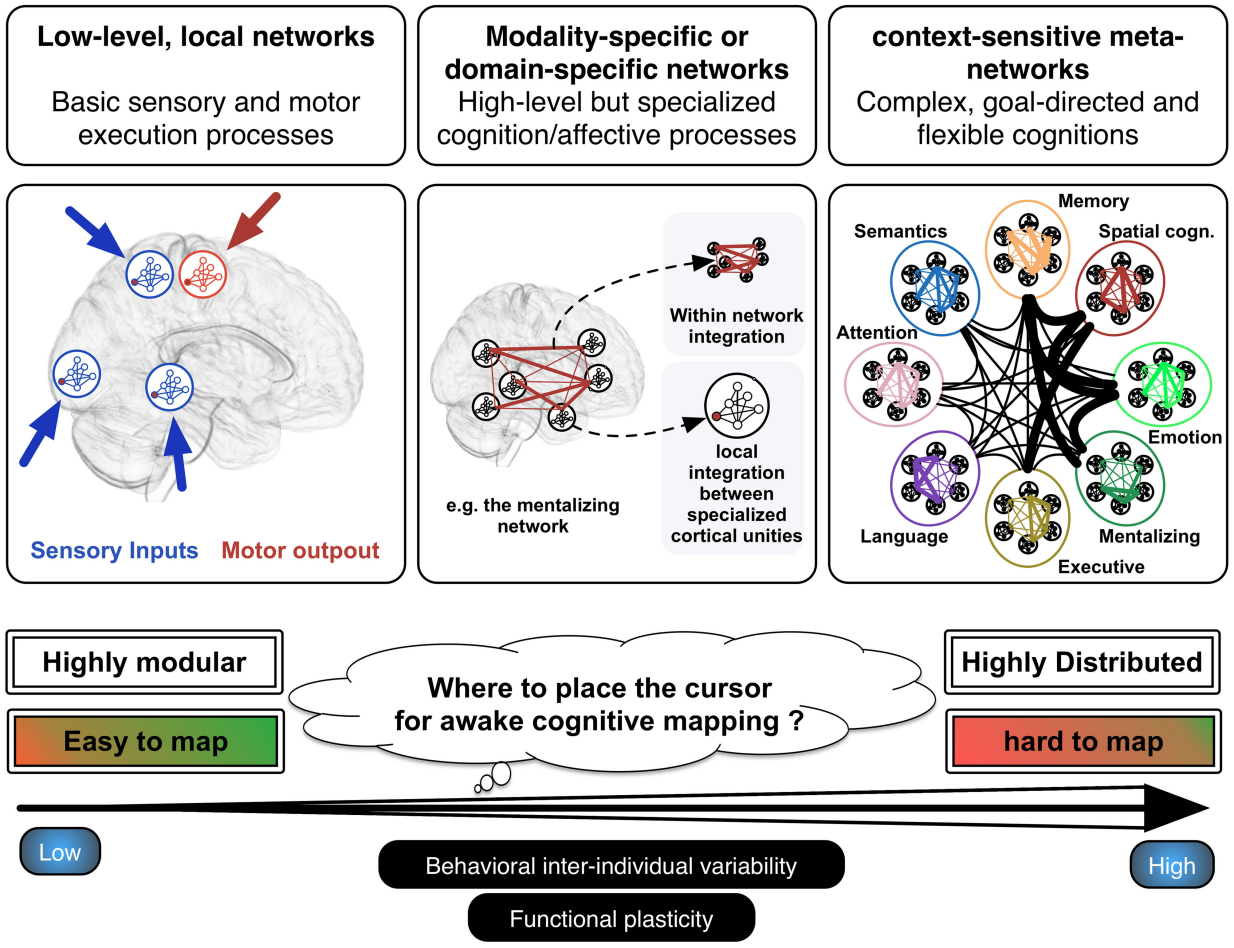
Hierarchical functioning of the anatomo-functional architecture. Basic sensorimotor processes are performed by low-level, local and highly modular networks, whereas modality-specific or domain-specific functions are rather underlain by distributed networks. The latter are composed of different cortical epicenters, the functional integration of which is permitted by anatomical connectivity. At a higher level, complex or flexible cognitions or behavior result from the instantiation of meta-networks, which consist in specific and unique patterns of cross-network integration transiently generated to reach the task demands. Adapted from Herbet and Duffau (16) with permission.

Within the hierarchical anatomo-functional architecture described above (from highly modular to highly distributed processing) (see Figure 1), I argue that electrostimulation mapping performs very efficiently to identify and spare neural systems associated with sensorimotor or modality-specific/domain-specific functions (e.g., language and semantic processes, visuo-spatial attention among many others). As a matter of fact, the rate of lasting and debilitating deficits has been considerably reduced, leading almost all patients to quickly resume a normal professional activity even in the event of incidental discovery (97%) (25). However, electrostimulation is somewhat limited in its ability to identify higher-order, complex and flexible cognitions. In my view, this limitation is mainly due to three reasons. First, flexible cognitions heavily rely on the resources of multiple large-scale networks working in synchrony. It thus remains to see the extent to which short electrostimulations with low intensity might disturb brain-wide processing and lead to complex behavioral impairments. We currently know from works combining electrostimulation mapping and functional connectivity analyses that positive stimulation sites can be considered as veritable gateways to domain-specific networks (26, 27), but the behavioral impact of disrupting cortical areas that interface with multiple networks is unknown. Some studies indicate that electrostimulation is able to transiently abolish aspects of self-awareness or external awareness (5, 28, 29), but it is unclear whether the physiological basis of these behavioral impairments is the brain inability to form transient metasystems. It is possible that the disruption of highly integrative white matter tracts (i.e., that project in several lobes such as the inferior fronto-occipital fasciculus) is more capable of disorganizing the way networks communicate. Alternatively, as multiple hubs are likely to synchronize during normal behavior, multi-focal electrostimulations (i.e., stimulations performed on two or several cortical hubs at the same time) might be a way to map more accurately complex functions. While this approach needs to be explored, it may be quite difficult to set up in the constraining context of neurosurgery. Moreover, its potential oncological benefit must be evaluated.

Second, with the exception of multitasking-like paradigms (typically, motor execution plus semantic association, or picture naming) or *n*-back-like task (strong cognitive load) the neuropsychological tasks employed in the operating theater are well-controlled but might be considered as reductionist because not necessitating strong cognitive requirements. Yet recent meta-analytic studies indicate that ecological, realistic (vs. highly controlled, reductionist) behavioral paradigms are associated with recurrent patterns of functional activations that overlap with numerous functional networks (sensorimotor, modality-specific, and domain-general), suggesting that flexible and complex behaviors triggered by lifelike situations result from the integration of distinct but cooperating networks (30). This is not without interest considering that, what we want ultimately for patients, is to maintain the best level of interactions with the everyday (including social) environment after surgery. However, the intraoperative cognitive mapping does not really accommodate with complex behavioral stimuli due to the constraints inherent to the surgical procedure (e.g., stimulation time, positioning constrain, and so one). Some adaptations are nevertheless possible, in particular varying the complexity of the materials used. In this situation, the neurocognitive system under scrutiny is necessarily up-regulated by domain-general networks (i.e., attention and cognitive control), increasing sensitivity. More broadly, this raises the question as to how more ecological but still controlled tasks can be constructed without losing interpretability (i.e., what is the precise impact of stimulation on the function probed by the task). This is of course central to maintaining the validity of cognitive monitoring.

Third, some studies have shown that the efficiency with which the brain is able to reconfigure its networks as a function of current cognitive demands is strongly predictive of behavioral output (31). This important interindividual variability implies that complex behaviors are likely to be more difficult to map with electrostimulation.

That said, it remains an open question whether the cognitive mapping should be pushed forward, keeping in mind the onco-functional balance. However, it may be not necessary given the possible high resilience of the distributed systems that flexible cognitions engage, especially in the context of conditions known to stimulate neuroplasticity such as slow-growing tumors (32).

## On the Resilience and Flexibility of Complex Functional Systems

Although it is established that the brain reorganizes over time in response to glioma infiltration (32, 33), the mechanistic aspects of this reactional plasticity remains poorly understood. Several patterns of functional remodeling has been described, including loco-regional, intra-hemispheric and inter-hemispheric/homotopic reorganization patterns [e.g., (34, 35)]. However, the different factors constraining these dynamic modulations are clearly not understood, even if several advances have been recently made thanks to serial stimulation mappings performed in patients with recurrent tumors (36). In particular, bulky (weakly diffusive) gliomas may favor peritumoral plasticity whereas widely diffuse tumors may cause brain-wide reorganization. On the other hand, it is likely that the more integrated the function is, the more resilient the dedicated network is, especially when the function is underlain by a neural system that is distributed in both cerebral hemispheres. For example, despite the central role of the anterior temporal structures within the semantic memory network, unilateral damage of this region in various pathophysiological conditions (including glioma) does not result in the severe impairments of semantic representations it might be expected. The current interpretation is that the absence of strict lateralization increases the robustness of this functional system, with a central role of homotopic areas (37). Beyond, it has been shown that neurocognitive networks resting on associative areas are especially prone to be functionally compensated (4).

In the context of dynamic metasystems, it is somewhat expected that damage to cortical hubs established to participate to between-system coordination may have widespread neuropsychological consequences because of their central position in the anatomo-functional architecture. This is the case, especially in patients with sudden lesion such as stroke or traumatic injury. In these patient populations, it is indeed shown that focal disruption of connector or highly participating hubs has dramatic effects on both global network dynamics (38) and the brain's ability to coordinate its networks (39), leading to multidomain cognitive impairments (40, 41). In patients with diffuse low-grade glioma, to the best of my knowledge there are no well-conducted studies specifically assessing the impact of the tumor on the brain's ability to generate meta-networks and the extent to which this may cause neuropsychological deficits. However, some works seem to indicate that this is not the case. For example, in the study by Herbet et al. (42) the surgical excision of the ventral precuneus/posterior cingulate, a cortical hub with highly connective properties (43, 44), was not associated with severe and multidomain impairments, suggesting that activities of such hubs can be redeployed. Admittedly, however, longitudinally designed and well powered studies are needed to confirm it.

## Conclusion

In this opinion article, my attempt was to fuel the debate on whether higher-order and complex cognitions can be appropriately mapped during awake surgery in view of the flexible and highly distributed neural architecture from which they emerge. From a network perspective, such functions are probably difficult (but not impossible) to map with a good reliability because they necessitate coordination of multiple networks and are associated with a high-level of inter-individual behavioral variability. Furthermore, they are more likely to be easily compensable compared to basic or domain-specific functions. From a neuro-oncological standpoint, however, no works have currently assessed in a longitudinal manner if complex and flexible cognitions/behaviors are impaired following neurosurgical procedures (knowing that they are not probed with routine neuropsychological tasks) and if these possible impairments are disabling in daily life. This is an important point to assess before we go any further.

Some of the challenges described in this article might be potentially mitigated by the combined use of other tools that allow to manipulate more complex materials, outside the operating theater. From this perspective, navigated transcranial magnetic stimulation (TMS) may offer good opportunities to identify the critical cortical structures [and perhaps the interconnected white matter pathways if diffusion tractography is combined; e.g., (45)] involved in high-level cognitive or social processes before the surgery is performed. It may also help evaluate the functional impact of stimulation (and possibly of surgical resections) on neural hubs that interface with multiple networks. To my knowledge, *n*TMS is to date only used with (most of the time language) stimuli which are classically employed during the intraoperative cognitive monitoring. On the other hand, task-based functional connectivity MRI may provide critical information on how efficiently complex networks reorganize in response to tumor invasion or on how the different networks engaged in a complex function up-modulate their activities to compensate the neural loss (46). Determining the physiological markers of resilient networks is of major importance in adjusting the surgical procedure.

## Author Contributions

GH wrote the manuscript.

## Conflict of Interest

The author declares that the research was conducted in the absence of any commercial or financial relationships that could be construed as a potential conflict of interest.
